# Prevalence and Correlates of Perceived Infertility in Ghana

**DOI:** 10.1111/sifp.12136

**Published:** 2020-09-22

**Authors:** Chelsea B. Polis, Easmon Otupiri, Michelle Hindin, Doris W. Chiu, Sarah C. Keogh, Cara Aidoo, Roderick Larsen‐Reindorf, Suzanne O. Bell

**Affiliations:** ^1^ Chelsea B. Polis, Guttmacher Institute New York NY; ^2^ Johns Hopkins Bloomberg School of Public Health Baltimore MD; ^3^ Doris W. Chiu, Sarah C. Keogh Guttmacher Institute New York NY; ^4^ Suzanne O. Bell, Johns Hopkins Bloomberg School of Public Health, Baltimore, MD. Easmon Otupiri, Cara Aidoo, Roderick Larsen‐Reindorf, School of Public Health, Kwame Nkrumah University of Science and Technology, Ghana. Michelle Hindin, Population Council New York NY

## Abstract

Perceived infertility is an understudied phenomenon in low‐ and middle‐income countries, where biomedical infertility can have severe consequences, particularly for women. We conducted a nationally representative survey of Ghanaian women, estimated the prevalence of and reasons for perceived infertility, and assessed factors associated with higher levels of perceived infertility using a partial proportional odds model. Among 4,070 women, 13 percent believed they were “very likely” to have difficulty getting pregnant when they wanted to, 21 percent believed this was “somewhat likely,” and 66 percent believed this was “not at all likely.” Reasons for perceived infertility varied by whether the respondent was currently seeking pregnancy. In multivariable analysis, several factors were associated with higher levels of perceived infertility, while unexpectedly, women who reported ever using contraception were less likely to report perceived infertility. Acknowledging the need to address infertility globally and understanding the role of perceived infertility are important components in supporting people's ability to decide whether and when to have children.

## BACKGROUND

In Ghana, rates of unintended pregnancy and induced abortion are high (Keogh et al. 2020), and although desired family size and total fertility rates have generally declined over the past several decades, contraceptive prevalence remains relatively low (Ghana Statistical Service (GSS), Ghana Health Service (GHS), and ICF International 2015; Ghana Statistical Service (GSS), Ghana Health Service (GHS), and ICF 2018; PMA2020/Ghana 2017). In 2017, 27 percent of currently married women aged 15–49 reported using a modern method of contraception, with an additional 6 percent reporting traditional method use (PMA2020/Ghana 2017). Among currently married women who do not desire pregnancy, less than half (46 percent) reported using modern contraception (PMA2020/Ghana 2017). In Ghana, fear of health concerns and side effects are top reasons for nonuse of modern contraception among women with unmet need (Staveteig 2016), with particular concern around irregular menstrual cycles (Hindin, McGough, and Adanu 2014; Staveteig 2017). These fears may in turn be linked with perceptions that menstrual changes lead to infertility (Atijosan, Adeyeye, and Ogungbayi 2019; Polis, Hussain, and Berry 2018).

Many factors are perceived by Ghanaians to cause infertility. A qualitative study in northern Ghana suggested that perceptions about the causes of infertility vary: urban residents with higher educational attainment more commonly noted biological causes, while rural residents more commonly cited supernatural causes (Tabong and Adongo 2013b). The most frequently stated biological causes were prior use of contraceptives (which was perceived as linked with a “promiscuous” lifestyle) (Tabong and Adongo 2013b). Other biological reasons included previous abortions (particularly those done by unqualified individuals), female genital mutilation, sexually transmitted infections, blocked fallopian tubes, and uterine fibroids. Multiple studies in Ghana (and elsewhere) have documented perceptions that contraception and/or abortion (Chipeta, Chimwaza, and Kalilani‐Phiri 2010; Wilkinson and Callister 2010; Tabong and Adongo 2013b; Teye 2013; Adongo et al. 2014; Hindin, McGough, and Adanu 2014; Krugu et al. 2017; Sedlander et al. 2018; Atijosan, Adeyeye, and Ogungbayi 2019) can lead to infertility in women, despite evidence that neither reversible contraceptive methods nor safe abortions adversely impact future fertility (Girum and Wasie 2018; Vayssière et al. 2018). Supernatural causes noted by participants included bewitching, pledges made to gods to accrue wealth in exchange for womanhood, or as punishment for masturbation or marital infidelity (Tabong and Adongo 2013b; Yaw Osei 2016). Infertility is clinically defined as failure to establish pregnancy after 12 months of regular, unprotected sexual intercourse (Zegers‐Hochschild et al. 2017), and perceived infertility has been defined as an individual's *belief* that they are unable to conceive, regardless of whether this belief is medically accurate (Polis and Zabin 2012). In parts of Ghana, having one child or no sons may also be viewed as a form of infertility (Tabong and Adongo 2013b).

Individuals who perceive themselves to be infertile may fear negative consequences stemming from the stigma of infertility. Studies in sub‐Saharan African countries and elsewhere find that stigma around infertility is pervasive and highly consequential (Rouchou 2013; Bornstein et al. 2020). In Ghana, as in many other countries, parenthood is viewed as a critical component for obtaining social status and respect (Tabong and Adongo 2013a, 2013b). Infertile Ghanaians—particularly women—may be subject to severe social, health, and economic consequences, including social ostracism (Alhassan, Ziblim, and Muntaka 2014; Anokye et al. 2017; Fledderjohann 2012; Tabong and Adongo 2013a), psychological distress (Alhassan, Ziblim, and Muntaka 2014; Anokye et al. 2017; Donkor, Naab, and Kussiwaah 2017; Fledderjohann 2012; Tabong and Adongo 2013b), reduced sexual satisfaction (Donkor, Naab, and Kussiwaah 2017; Nyarko and Amu 2015), relationship difficulties, marital instability and increased polygyny (Anokye et al. 2017; Fledderjohann 2012, 2017; Nyarko and Amu 2015; Tabong and Adongo 2013a; Wilkinson and Callister 2010), strained relationships with extended family members (Tabong and Adongo 2013a; Wilkinson and Callister 2010), and a loss of access to status and wealth (Tabong and Adongo 2013a; Alhassan, Ziblim, and Muntaka 2014). Though some infertile women may adopt children of relatives, in some parts of Ghana, infertile women may be labeled as witches, abandoned by relatives, forbidden from interacting with children, or denied hereditary chieftaincy status (Tabong and Adongo 2013a). Although infertility is generally blamed on women, men perceived to be infertile may also experience negative impacts, such as social ostracism, exclusion from community leadership roles, and for some, rituals requiring another man to impregnate his wife (Fledderjohann 2012; Tabong and Adongo 2013a). Childless couples may be denied membership in the ancestral world, as certain funerary rites must be performed by children of the deceased (Tabong and Adongo 2013a). Anticipation of adverse consequences of infertility may impact mental health or prompt engagement in higher risk sexual behaviors to fulfill social pressures to conceive (Dhont et al. 2011), and stress may even play a role in reducing actual fecundability (Wesselink et al. 2018). Fertility perceptions can also impact other health‐related behaviors, including contraceptive use. If someone perceives that they are not at risk of conceiving, they may forego contraceptive use, even if they are not seeking pregnancy (Gemmill 2018). If that individual is not infertile, they may therefore be exposed to risk of unintended pregnancy.

Infertility has been a neglected issue in public health, particularly in low‐ and middle‐income countries (LMICs) (Gerrits et al. 2017). Even fewer studies in LMICs have examined perceived infertility (Fledderjohann and Johnson 2016; Polis et al. 2020b), despite possible negative impacts of a perceived potential failure to conceive among those who desire a child, and the increased risk of unintended pregnancy among those seeking to avoid pregnancy but not using contraception. Exploratory research is needed to understand the magnitude and correlates of perceived infertility, defined in this paper as heightened concerns that getting pregnant when desired may be difficult. We estimated the prevalence of perceived infertility in a nationally representative sample of Ghanaian women aged 15–49, using a measure similar to one used among young adults in southern Malawi (Polis et al. 2020b) and the United States (Polis and Zabin 2012). We also investigated reasons for infertility‐related perceptions, examined characteristics associated with these perceptions, and used hypothetical vignettes to assess understandings about the likelihood of pregnancy in various scenarios.

## METHODS

### Survey Design and Sampling

We conducted a nationally representative, community‐based survey of reproductive age women in Ghana as part of a larger study on abortion incidence in Ghana in 2018 (Keogh et al. 2020). Kwame Nkrumah University of Science and Technology (KNUST) conducted fieldwork in 2018, with technical support from the Guttmacher Institute and the Performance Monitoring and Accountability 2020 team at the Johns Hopkins Bloomberg School of Public Health.

We used a multistage stratified cluster sampling design. Using probability‐proportional‐to‐size sampling, the Ghana Statistical Service randomly selected 100 enumeration areas (which hold about 200 households each) across 20 sampling strata determined using the 10 administrative regions and urban–rural location. We listed, mapped, and randomly selected 42 households in each selected enumeration area. In selected households, we conducted a household survey to collect socioeconomic information and to identify eligible female respondents aged 15–49 who stayed in the household the night before the interview. We invited all eligible women in each of the 4,200 selected households to give informed consent and participate in the full survey. We collected 4,123 completed household forms, and of those, 534 households had no eligible participant. Among 4,754 eligible women aged 15–49 whom we identified, we successfully interviewed 4,722 (99 percent response rate). Participants who completed the full survey received a bar of soap.

The full survey included modules with various approaches to estimate abortion, as well as modules on sociodemographics, fertility preferences and experiences, contraceptive use and experiences, and knowledge of fertility and perceived infertility. We identified differences on zone and marital status in our sample versus a larger sample from the 2017 Ghana Maternal Health Survey (GMHS) (Ghana Statistical Service (GSS), Ghana Health Service (GHS), and ICF 2018). Thus, we used poststratification weights to ensure our sample was comparable to the GMHS sample.

Trained resident enumerators (REs), typically women over age 21 who are familiar with the language and culture of the region and have at least a high school diploma, served as data collectors for the project. Training included establishing consensus on translation of the survey instrument, as there are 49 languages spoken in Ghana (many of which are unwritten). REs administered structured questionnaires face‐to‐face in a private area using an Android smartphone enabled with Open Data Kit electronic data collection software. Interviews were conducted in English or the respondent's local language. We estimate that the full survey, uninterrupted, took approximately one hour and 15 minutes, on average. We obtained ethical approval from Institutional Review Boards of the Guttmacher Institute, the Johns Hopkins Bloomberg School of Public Health, and the KNUST Committee on Human Research, Publication and Ethics.

For this analysis, we excluded those who reported that they (or their partner) were currently sterilized (n = 47), were currently pregnant (n = 359), never had a menstrual period (n = 23), or were menopausal or had a hysterectomy (n = 75). We also excluded 146 women who responded “do not know” or declined to answer the key question on perceived infertility, and two women who declined to indicate if they were currently trying to conceive. We conducted our analysis among the remaining 4,070 participants.

### Variables

We derived our dependent variable from the following question on perceived infertility: “Some people are unable to become pregnant, even if they want to. Do you think it is not at all likely, somewhat likely, or very likely that you will have difficulty getting pregnant when you want to in the future?” In addition to these response options, we noted if respondents answered that they did not know or if they provided no response. Women who responded “somewhat” or “very” likely to the question were asked about the reason(s) why they believed they would have difficulty getting pregnant in the future, and interviewers classified those responses into one of 12 predetermined categories (as listed in Table 1), including an “other” option.

**TABLE 1:** Weighted counts and percentages by selected characteristics

Characteristic	Weighted N(N = 4,124)	Percentage
Women's demographic characteristics		
Age		
15–19	803	19
20–29	1,382	34
30–39	1,212	29
40–49	727	18
Highest level of school attained		
Attended no school	538	13
Attended primary or middle	2,404	58
Attended secondary or more	1,183	29
Religion		
Any Christian	2,991	73
Muslim	564	14
Traditional/other	339	8
No religion	229	6
Ethnicity		
Akan	1,990	48
Ga/Dangme	288	7
Ewe	674	16
Guan	37	1
Mole‐Dagbani	179	4
Grusi	59	1
Gurma	66	2
Mande	2	0
Other	827	20
Household characteristics		
Ecological zone		
Northern	510	12
Middle	1,906	46
Coastal	1,708	41
Urban/rural residence		
Urban	2,448	59
Rural	1,676	41
Wealth		
Poorest 60% (among all survey respondents)	2,250	55
Richest 40% (among all survey respondents)	1,844	45
Sexual and reproductive health experiences and characteristics		
Union status		
Currently married/cohabiting	2,252	55
Formerly married/cohabiting	629	15
Never married/cohabiting	1,233	30
Sexual experience and recency		
Never had sex	613	15
Sex in last three months	2,456	60
Sex >3 months ago	1,045	25
Pregnancy/birth history		
Never pregnant	1,179	29
Ever pregnant, no birth	200	5
Ever pregnant, had birth	2,718	66
Parity		
No kids or never pregnant	1,382	34
1–4 born	2,118	52
4+ born	597	15
Currently trying for pregnancy		
No	3,572	87
Yes	552	13
Ever use of contraception		
Only ever traditional method(s)	204	5
Ever hormonal/LARC method(s)	1,487	37
Ever nonhormonal modern method(s) or EC (and never use of hormonal/LARC methods)	462	11
Never used contraception	1,883	47
Current contraceptive use		
Only traditional method(s)	204	5
LARC/hormonal method(s)	710	18
Non‐hormonal modern method(s) or EC	257	7
Not a current user	2,756	70
Correctly identified when during the menstrual cycle pregnancy is most likely		
DK/NR/incorrect timing	2,710	66
Knows most fertile days	1,414	34
Reported ever miscarriage		
No miscarriage reported	3,370	82
Miscarriage reported	754	18
Reported ever inducing abortion		
No induced abortion reported	3,257	79
Induced abortion reported	861	21
Time since last menstruation		
Within last two months	3,525	85
>2 months ago	421	10
Before last birth	177	4
Likelihood of sex without contraception in next three months		
Not at all likely	1,716	42
Somewhat likely	357	9
Very likely	946	23
No sex expected in next three months	1,079	26
Do not know	22	1
Perceived likelihood of having difficulty getting pregnant when desired		
Not at all likely	2,704	66
Somewhat likely	880	21
Very likely	540	13

For the independent variables, we examined variables identified as associated with perceived infertility in prior, similar studies (age, education, ethnicity, sexual experience, pregnancy/birth history, parity, currently trying for pregnancy, current contraceptive use, report of ever experiencing miscarriage, and report of ever inducing abortion) (Polis and Zabin 2012; Polis et al. 2020b). We also considered other variables that we theorized *a priori* may be relevant in the Ghanaian context (ecological zone, union status, urban/rural residence, religion, wealth, ever use of contraception, knowledge of when during the menstrual cycle pregnancy is most likely, and time since last menstruation).

We coded age and parity categorically in descriptive analysis and continuously in regressions. We treated all other demographic, household, and sexual and reproductive health experiences and characteristics variables as categorical. We classified contraceptive methods as traditional (rhythm, withdrawal, washing, Primolut N1Primolut N (or “N‐tablet”) is a pill containing 5 mg of synthetic progesterone, intended for use in regulating menstrual cycles, dysmenorrhea, or endometriosis. In Ghana, N‐tablet is sometimes misused as contraception or emergency contraception., or other traditional), hormonal (implants, three‐month or one‐month injectable, pill) or nonhormonal modern methods (intrauterine devices [IUDs], male condoms, female condoms, diaphragms, foam, Standard Days Method/Cycle Beads, or lactational amenorrhea method). Individuals who stated that they had ever used but were not currently using female sterilization (n = 19) or both male and female sterilization (n = 21) were retained in an analysis and included in the nonhormonal modern methods group. The groupings for modern methods are premised on the fact that people may view methods that do not involve sustained use of hormones differently (with respect to the potential to lead to infertility) than methods that do not contain hormones or only involve hormones used episodically. We included emergency contraception (EC) in the nonhormonal modern category as we were interested in whether use of hormonal methods used in an ongoing manner (versus episodically, as with EC) impacted perceived infertility. As some studies in Ghana note routine use of EC (Chin‐Quee et al. 2012), we also conducted a sensitivity analysis to see if results changed by including EC in the hormonal category. We created a categorical variable based on a series of questions to indicate whether women correctly identified the period of highest fecundability (“halfway between two periods”). We also explored how likely respondents anticipated it to be that they would have sex without any contraceptive within the subsequent three months, and if so, why they anticipated doing this. Given that a very small proportion (3 percent) of these respondents stated that perceived infertility was the reason they expected to have sex without contraception in the near future, this variable was not considered for inclusion in multivariable models.

We asked all participants about the likelihood of pregnancy (not at all, somewhat, or very likely to get pregnant) under various hypothetical vignettes describing women of different age categories and reproductive health‐related experiences. For example, vignettes included questions pertaining to pregnancy after abortion, contraceptive nonuse without becoming pregnant, injectable‐induced amenorrhea, IUD removal, and for a woman potentially nearing the end of her reproductive years.

### Data Analysis

We performed analyses in Stata version 16, (StataCorp 2019) using *svy* commands to account for the complex sampling design, with the *subpop* option to restrict to our analytic population. We calculated weighted counts and percentages of respondents for each characteristic and for likelihood of pregnancy vignettes. We initially constructed bivariate and multivariable generalized ordered logistic regression models to identify factors associated with higher odds of a woman believing she would have difficulty getting pregnant when she wanted to in the future (“perceived infertility”). Standard ordinal models require that all independent variables meet the proportional odds assumption (POA); in other words, that if we fit two sets of binary logistic regressions (not likely vs. somewhat/very likely; not/somewhat likely vs. very likely), a common odds ratio would be observed across both regressions for each independent variable. Some of our independent variables violated the POA, so a standard ordinal model would be inappropriate. Instead, we estimated a partial proportional odds model (Williams 2006, 2016), which relaxes the requirement that all independent variables meet the POA, and generates results more parsimonious and interpretable than those from a multinomial model. We used the *autofit* option in the *gologit2* package to assess if the POA held for each independent variable, based on Wald tests using *α* < 0.025. We used this more stringent significance level in assessing for POA violations to reduce the likelihood we would detect violations by chance alone (Williams 2006). For independent variables that met the POA, we present one adjusted odds ratio (adjOR), interpretable as in a standard ordinal regression model (described above). For independent variables that violated the POA, we report two adjORs (one for not likely vs. somewhat/very likely and another for not/somewhat likely vs. very likely to have difficulty becoming pregnant when desired).

We did not perform any imputation, as missingness was 2 percent or less on all variables considered. To avoid estimation issues, we did not consider including variables that had small (n < 5) cell sizes when cross‐tabulated with the perceived infertility variable (this was only the case with our ethnicity variable). In lieu of ethnicity, we considered zone (which is correlated with ethnicity) for inclusion. We also excluded current contraceptive use due to collinearity with ever contraceptive use, which we determined to be a better representation of a potential antecedent to perceived infertility. We decided *a priori* to retain age and education regardless of statistical significance. From these theoretically related variables, we identified the most parsimonious models by assessing changes in Akaike Information Criterion (AIC) and Bayes Information Criterion (BIC) when variables were removed from the model, using the *fitstat* command (Long and Freese 2014). AIC and BIC are not estimable when using *svy* commands in Stata, so we did not use *svy* commands when comparing models. We also considered polynomial transformations of continuous variables to allow for potential curvilinear effects, and assessed if there was an interaction between age and education, as observed in a prior study (Polis et al. 2020b).

The study funders had no role in study design; collection, analysis, or interpretation of the data; or in the writing or decision to submit the report.

## RESULTS

### Descriptive Analysis

Slightly over half (53 percent) of respondents were under 30 years (average age 29.2 years), and a majority (58 percent) had attended primary or middle school, while 29 percent had attended secondary school or more (Table 1). Nearly three‐quarters (73 percent) of respondents identified as Christian, nearly half (48 percent) were of Akan ethnicity, and 59 percent lived in an urban area. Nearly one‐third (30 percent) of respondents had never been married, and 15 percent never had sexual intercourse. One‐third (34 percent) of respondents had no children and about half (52 percent) had delivered between 1 and 4 children. About 13 percent of respondents were currently trying to become pregnant. Nearly half of our sample (47 percent) reported never having used a contraceptive method, 5 percent reported only ever having used a traditional method(s), 37 percent ever used a hormonal or long‐acting reversible contraception (LARC) method(s), and the remaining 11 percent ever used a nonhormonal modern method(s) and/or EC. Current contraceptive use was lower: 70 percent reported not using any method(s), 5 percent reported using a traditional method(s), 18 percent reported using a hormonal method(s) or LARC, and 7 percent reported using a nonhormonal modern method(s) and/or EC. Approximately one in five women reported ever having a miscarriage (18 percent) or inducing an abortion (21 percent). About one‐third (34 percent) of women were correctly identified when during the menstrual cycle conception is most likely. Most (85 percent) women had menstruated within the last two months.

Nearly one‐quarter of women (23 percent) felt it was “very likely” that they would have sex without using contraception in the next three months, and an additional 9 percent believed this was “somewhat likely.” However, when asked the main reason for this expectation, very few women gave reasons pertaining to fertility perceptions: only 3 percent said “I believe myself or my partner is unable to become pregnant” (data not shown).

### Prevalence of Perceived Infertility

Overall, 13 percent of all women believed they were “very likely,” 21 percent believed they were “somewhat likely,” and 66 percent believed they were “not at all likely” to have difficulty getting pregnant when desired (Table 1). When restricted to women who affirmed a desire to have a/additional child(ren), these proportions remained similar (14 percent “very likely,” 22 percent “somewhat likely,” and 64 percent “not at all likely”; data not shown). Women aged 30 or older expressed significantly greater concern about perceived infertility: the proportion responding “very likely” was 10 percent among women under 30 and 17 percent among women aged 30 and older, p = 0.00; data not shown).

### Reasons for Perceived Infertility

Among the subset of participants (weighted N = 540) who indicated feeling “very likely” to have difficulty getting pregnant when desired, the top four reasons for this feeling included: age‐related concerns like menopause (24 percent), “other” reasons (not defined) (24 percent), having tried unsuccessfully to conceive for a year or more (24 percent), and having had sex without contraception and not conceiving, leading to a belief that she will never conceive (23 percent) (Table 2). Women not currently trying to conceive were more likely to note age‐related or “other” concerns; and women currently trying to conceive were more likely to note having tried to conceive unsuccessfully for a year or more or having had sex without contraception and not conceiving, leading to a belief that she will never conceive. As expected, age‐related concerns were significantly higher (p = 0.00) among women 40 or older (42 percent) compared to women under 40 (17 percent) (data not shown).

**TABLE 2:** Reasons for expecting difficulty getting pregnant to be “very likely," by current pregnancy intention

	All respondents (weighted N = 540)		Respondents not currently trying to conceive (weighted N = 407)		Respondents currently trying to conceive (weighted N = 133)		
Reason for belief	N	Percentage	N	Percentage	N	Percentage	p‐Value
Age‐related concerns such as menopause	131	24	111	28	20	15	0.01
“Other” reasons (undefined)	131	24	111	28	19	15	0.02
Having tried unsuccessfully to conceive for a year or more	130	24	71	18	59	44	0.01
Having had sex without contraception and not conceiving, leading to a belief that she will never conceive	123	23	71	18	52	39	0.00
Currently or previously using a contraceptive method that she believes made her unable to conceive	59	11	49	12	10	8	0.15
Having a medical condition or having undergone another medical procedure, which she believes made her unable to conceive	44	8	33	8	11	8	0.96
Health care provider told her she might have difficulty conceiving	39	7	30	7	9	7	0.85
Irregular menstrual cycle	35	7	28	7	7	6	0.58
Superstition	35	7	27	7	8	6	0.77
Had an abortion, which she believes made her unable to conceive	30	6	21	5	10	7	0.50
Other people in my family are unable to conceive	24	5	21	5	3	2	0.22
Lack of sperm retention	12	2	7	2	5	4	0.07

NOTE: All N's and percentages are weighted. Totals do not sum to 100% because respondents could select more than one response. Interviewers did not read response options; women responded and interviewers selected the most applicable category.

Less commonly noted reasons included: currently or previously using a contraceptive method, which she believes made her unable to conceive (11 percent); having a medical condition or having undergone a medical procedure that she believes made her unable to conceive (8 percent); health care provider told her she might have difficulty conceiving (7 percent); irregular menstrual cycle (7 percent); superstition (7 percent); having had an abortion (6 percent); having other people in her family who are unable to conceive (5 percent); and lack of sperm retention (2 percent). The proportion noting these reasons did not differ significantly by pregnancy‐trying status.

### Factors Associated with Higher Levels of Perceived Infertility

We identified several factors associated with higher levels of perceived infertility (described below as “perceived infertility” for brevity). In bivariate regression, the following characteristics were significantly associated (p‐value for Wald test <0.05) with perceived infertility: older age, lower level of school attainment, living in the Middle (vs. Coastal) zone (only for the not vs. somewhat/very likely comparison), being poorer (only for the not vs. somewhat/very likely comparison), being currently or formerly married or cohabitating, higher parity and parity squared, currently trying to become pregnant, never having used contraception, and having self‐reported past miscarriage (Table 3). The following variables were also significantly associated with perceived infertility in bivariate analysis, but did not contribute to the final multivariable model: having ever had sexual intercourse, having ever given birth, having incorrect knowledge of the most fertile days of the menstrual cycle, and having last menstruated over two months ago (data not shown).

**TABLE 3:** Unadjusted and adjusted odds ratios and 95% CIs for expected likelihood of difficulty getting pregnant when respondent wants to in the future

	Unadjusted ORs and 95% CIs		Adjusted ORs and 95% CIs	
	Somewhat/very likely^a^ versus not likely^a^	Very likely^a^ versus not/somewhat likely^a^	Somewhat/very likely^a^ versus not likely^a^	Very likely^a^ versus not/somewhat likely^a^
	OR (95% CI)	OR (95% CI)	AdjOR (95% CI)	AdjOR (95% CI)
Age (continuous)	***1.03*** (*1.02–1.05*)		***1.03*** (*1.01–1.05*)	
Highest level of school attained (ref: secondary or more)				
No school	***2.04*** (*1.37–3.04*)		*1.14* (*0.81–1.60*)	
Primary or middle	***1.78*** (*1.35–2.34*)		***1.29*** (*1.02–1.64*)	
Ecological zone (ref: Coastal)				
Northern	*1.76* (*0.92–3.36*)		*1.38* (*0.71–2.66*)	
Middle	**2.37** (1.36*–*4.14)	1.06 (0.55*–*2.03)	**2.27 (**1.29*–*3.99)	1.01 (0.54*–*1.89)
Wealth (ref: richest 40%)				
Poorest 60%	**1.89** (1.32*–*2.71)	1.26 (0.83*–*1.91)	**1.54 (**1.10*–*2.16)	1.08 (0.76*–*1.54)
Union status (ref: never married/cohabitating)				
Currently married/cohabiting	***2.20*** (*1.70–2.85*)		***1.85*** (*1.35–2.55*)	
Formerly married/cohabitating	***1.90*** (*1.33–2.73*)		***1.55*** (*1.05–2.30*)	
Parity (continuous)	***1.12*** (*1.07–1.18*)		***0.90*** (*0.83–0.99*)	
Parity squared (continuous)	***1.02*** (*1.00–1.03*)		***1.02*** (*1.01–1.03*)	
Currently trying to become pregnant (ref: not trying)				
Trying	***2.10*** (*1.57–2.82*)		***1.78*** (*1.29–2.45*)	
Ever use of contraception (ref: never used contraception)				
Only ever traditional method(s)	*0.75* (*0.48–1.18*)		***0.62*** (*0.39–0.96*)	
Ever hormonal/LARC method(s)	*0.88* (*0.73–1.07*)		***0.72*** (*0.59–0.88*)	
Ever nonhormonal modern method(s) or EC (and never use of hormonal/LARC)	***0.47*** (*0.34–0.64*)		***0.55*** (*0.40–0.75*)	
Reported ever miscarriage (ref: no)				
Yes	***1.70*** (*1.39–2.07*)		***1.34*** (*1.10–1.62*)	
Reported ever induced abortion (ref: no)
Yes	*1.02* (*0.83–1.26*)		*1.05* (*0.83–1.32*)	

NOTE: Odds ratios and 95% CIs in italics are for variables that met the proportional odds assumption, and are interpretable as in an ordinal regression. For those that violated the proportional odds assumption, we report two adjORs (one for not likely vs. somewhat/very likely, another for not/somewhat likely vs. very likely to have difficulty becoming pregnant when desired). ORs and adjORs shown in bold were statistically significant.

^a^ To have difficulty getting pregnant when you want to in the future.

In multivariable analysis, several characteristics remained independently associated with higher odds of perceived infertility (Table 3). Each one‐year increase in age corresponded to a significant 3 percent increase in odds of perceived infertility (adjOR: 1.03, 95 percent CI: 1.01–1.05). Compared to women with secondary or higher education, women who completed primary or middle school had a 29 percent increase in the odds of perceived infertility (adjOR: 1.29, 95 percent CI: 1.02–1.64). Living in the Middle zone (vs. the Coastal zone) yielded over twice the odds of responding somewhat or very likely (vs. not at all likely) to the question on perceived infertility (adjOR: 2.27, 95 percent CI: 1.29–3.99), but did not distinguish between the highest level of perceived infertility (very likely) and being not at all or somewhat likely. A similar pattern was observed for poorer women, who had 54 percent higher odds of responding somewhat or very likely (vs. not at all likely) to the question on perceived infertility (adjOR: 1.54, 95 percent CI: 1.10–2.16). Currently and formerly married or cohabitating women had higher odds of perceived infertility (adjOR: 1.85, 95 percent CI: 1.35–2.55, and adjOR: 1.55, 95 percent CI: 1.05–2.30, respectively) as compared with women who were never married or cohabitating. Parity had a U‐shaped relationship with perceived infertility: each additional child born initially decreased the odds of perceived infertility by 10 percent (adjOR: 0.90, 95 percent CI: 0.83–0.99) but at the highest levels of parity, this relationship was inverted, with odds of perceived infertility increasing by 2 percent for each additional child (adjOR: 1.02, 95 percent CI: 1.01–1.03). Women currently trying to become pregnant had 78 percent higher odds of perceived infertility (adjOR: 1.78, 95 percent CI: 1.29–2.45), and women who reported ever experiencing miscarriage had 34 percent higher odds (adjOR: 1.34, 95 percent CI: 1.10–1.62), whereas having reported ever inducing abortion was not significantly associated with higher levels of perceived infertility (adjOR: 1.05, 95 percent CI: 0.83–1.32). Compared to women who had never used contraception, we observed lower odds of perceived infertility among women who had ever used a traditional method(s) (adjOR: 0.62, 95 percent CI: 0.39–0.96), nonhormonal modern method(s) or EC (adjOR: 0.55, 95 percent CI: 0.40–0.75), or hormonal or LARC method(s) (adjOR: 0.72, 95 percent CI: 0.59–0.88), and results were similar if we grouped EC with other hormonal methods (data not shown).

### Perceptions of the Likelihood of Pregnancy in Hypothetical Vignettes

In all hypothetical vignettes except the one pertaining to older age, over half of participants believed the woman described was “very likely” to get pregnant within a specified time frame that varied based on scenario (see Table 4 for full descriptions). Respondents held the least concern about future fertility for an 18‐year‐old woman undergoing abortion with a qualified doctor (only 4 percent not at all likely and 25 percent somewhat likely to become pregnant in the next year) or for a 20‐year‐old woman who had sex without contraception for four months without becoming pregnant (only 7 percent not at all and 28 percent somewhat likely to become pregnant in the next year). Concern was slightly higher for a 27‐year‐old woman experiencing injectable‐induced amenorrhea (10 percent not at all likely, and 33 percent somewhat likely to become pregnant in the next two years) or for a 32‐year‐old woman with two children who had an IUD removed (7 percent not at all likely and 35 percent somewhat likely to become pregnant in the next year). Concern was highest for a 43‐year‐old woman with two children who is not using contraception (18 percent not at all and 44 percent somewhat likely to get pregnant in the next year).

**TABLE 4:** Perceptions of pregnancy likelihood under hypothetical scenarios

Perceived likelihood of pregnancy			
Scenario	Not at all likely to get pregnant	Somewhat likely to get pregnant	Very likely to get pregnant
An 18‐year‐old woman in your community just terminated a pregnancy at a facility with a qualified doctor. How likely do you think it is that she can become pregnant within a year if she wants to?	4%	25%	71%
A 20‐year‐old woman in your community has had sex without using any form of contraception for four months without becoming pregnant. How likely do you think it is that she can become pregnant within a year if she wants to?	7%	28%	65%
A 27‐year‐old woman in your community is using injectables, and has not been bleeding during the past year. She plans to stop using injectables this month, and wants to try to become pregnant within the next two years. How likely do you think it is that she can become pregnant within the next two years?	10%	33%	57%
A 32‐year‐old woman in your community has two children, aged 6 and 3. After her 3‐year‐old was born, she began using an IUD. Last month, she had the IUD removed. How likely do you think it is that she can become pregnant within a year if she wants to?	7%	35%	58%
A 43‐year‐old woman in your community has two children and is not using contraception. How likely do you think it is that she can become pregnant if she wants to?	18%	44%	38%

## DISCUSSION

Perceived infertility among reproductive age women in Ghana is not uncommon, but is neither as rare nor as widespread as in other contexts where it has been measured using a similar instrument. Overall, 13 percent of Ghanaian women thought they would be very likely to experience difficulty getting pregnant when desired, with an additional 21 percent reporting they thought this scenario was somewhat likely. The proportion of Ghanaian women of comparable ages who responded with the greatest level of concern about perceived infertility was approximately half that of American women aged 18–29 years (10 percent vs. 19 percent) and approximately double that of southern Malawian women aged 21–29 years (11 percent vs. 5 percent) (Polis et al. 2020b; Polis and Zabin 2012). Unsurprisingly, perceived infertility was higher among older women in our study. This finding was bolstered by the fact that age was among the most common reasons for concern about future fertility, by the finding that each additional year of age was associated with higher odds of perceived infertility, and by the strong role of age in responses to hypothetical vignettes on pregnancy probabilities.

Our study illuminated some common reasons for perceived infertility among Ghanaian women (age, unsuccessful prior attempts to conceive, having sex without contraception without conceiving), however, a substantial proportion of women selected the “other” response option (indicating that our precoded categories did not fully capture all potential reasons). A recent study in southern Malawi found that past or present contraceptive use, medical concerns, and no prior pregnancies were among the top reasons provided in open‐ended responses by women to explain their perceived infertility (Polis et al. 2020b). Qualitative studies among women in LMICs could help more fully characterize the array of reasons for perceived infertility.

Prior experience using contraception played a more limited role than expected, even for methods expected to cause irregular bleeding patterns. Only 11 percent of women who felt they were “very” likely to have difficulty getting pregnant when desired linked this belief to current or previous contraceptive use. In hypothetical pregnancy vignettes, the majority of respondents (57–58 percent) believed that women in the scenarios were “very likely to get pregnant” despite past use of injectables (and associated amenorrhea) and IUDs. Most strikingly, in our study, women who reported ever using any method of contraception were—paradoxically—*less* likely to report perceived infertility. It is unclear if perceptions about the potential impact of contraceptives on future fertility in Ghana are less extreme than previously suggested (Tabong and Adongo 2013b; Adongo et al. 2014; Hindin, McGough, and Adanu 2014; Krugu et al. 2017) or have been mitigated during recent years, if the duration of time since discontinuation blunts any such concerns, or if those most predisposed to worry about such concerns are less likely to ever initiate any method of contraception. In these cross‐sectional data, we could not assess whether perceived infertility influenced subsequent contraceptive use. As in a prior analysis in the United States (Polis and Zabin 2012), we asked respondents with perceived infertility if and why they expected to have sex without any form of contraception in the next few months. Examination of the reasons given suggested that this variable would be a poor proxy in Ghana for subsequent contraceptive nonuse due to perceived infertility; longitudinal data are needed.

A qualitative study in Ghana noted strong concerns about the potential effect of medication abortion on fertility (Tabong and Adongo 2013b). Perceptions regarding the potential impact of abortion on future fertility may have changed over time; in our study, prior experience of miscarriage was associated with higher odds of perceived infertility, while induced abortion was not. This unexpected finding is supported elsewhere in our data, for example, abortion was not a common reason given for perceived infertility, and in the hypothetical vignettes, abortion by a qualified provider did not appear to induce a great deal of concern about future fertility for a young woman. However, an estimated 71 percent of all abortions in Ghana are provided by unapproved providers and/or in unapproved facilities (Polis et al. 2020a), and our vignettes did not inquire about how such abortions may impact fertility. Furthermore, some women who experienced induced abortion may have felt more comfortable reporting this experience as a miscarriage, due to social desirability bias. Additional research investigating current perceptions among Ghanaian women regarding the impact of abortion on fertility, by abortion type and provider type, could clarify discrepancies between our findings and those in other studies. Other factors that appeared to play a limited role included medical conditions/procedures, health care providers indicating a potential for difficulty conceiving, irregular menstrual cycles, superstition, familial infertility, or lack of sperm retention.

Unsurprisingly, the odds of perceived infertility were nearly twice as high among women trying to become pregnant around the time of the survey, who may be more actively focused on ensuring their ability to conceive. Although we did not collect information on the duration of trying time, these perceptions may be related to current difficulties conceiving (and potentially more predictive of clinical infertility).

To date, most data on perceived infertility derive from the United States, where multiple studies have shown that common reasons for reporting contraceptive nonuse is a belief that pregnancy is unlikely or impossible (Gemmill 2018; Frohwirth, Moore, and Maniaci 2013; Mosher, Jones, and Abma 2015; Cabral et al. 2018), and where a longitudinal study suggested that perceived infertility was associated with contraceptive nonuse one year later (Gemmill 2018). Fewer studies have examined perceived infertility in LMICs (Barden‐O'Fallon 2005; Fledderjohann and Johnson 2016; Leonard 2002; Polis et al. 2020b; Rao et al. 2018). In the only related study in Ghana we are aware of, Fledderjohann and Johnson (2016) examined self‐assessed difficulties conceiving by asking married or in union women in six communities how long it generally takes them to become pregnant, and found some evidence that these perceptions are aligned with clinically defined infertility. In their sample, 32 percent responded that it “takes a long time” and 6 percent said that they “can no longer become pregnant.” Although this approach allows individuals to define for themselves if waiting time to pregnancy has been “long” (which may be particularly important for examining social consequences of perceived infertility), it is limited in its applicability to individuals who have not previously attempted pregnancy, and complicates comparing perceived infertility prevalence across cultures and geographies. Fledderjohann and Johnson (2016) call for research to consider a variety of measures assessing perceived difficulties conceiving, a gap the present study helps to address.

This study has several additional strengths, including being the first quantitative study to explore perceived infertility in Ghana using a large, nationally representative sample of reproductive‐age women, and the first to include women aged 30 and older. We used similar measures of perceived infertility as in other settings, enabling cross‐national comparisons. Our analysis included data on socioeconomic and reproductive factors associated with perceived infertility in prior studies, and the large dataset enabled detailed investigation of multiple factors. We employed multiple approaches to assess beliefs about fertility, while making efforts to minimize response biases. For example, based on input from Ghanaian researchers, we adapted our key questions in a culturally informed manner (without losing the meaning or purpose of the question), to reduce the likelihood of responses being influenced by social norms that stigmatize infertility.

This study has limitations. First, we were not able to examine perceived infertility in men, nor did our questionnaire collect information on the proportion of respondents in polygynous relationships. The 2017 GMHS suggests that among Ghanaian women in Union, 12 percent have a husband or partner with one co‐wife, and an additional 2 percent have two or more co‐wives. Second, our study was not designed to enable quantification of the relationship between perceived infertility and actual infertility, nor did we include a measure of perceived health status. Third, we had limited success in assessing specific reasons for perceptions of infertility; qualitative studies are warranted. Fourth, our key question does not distinguish between respondents who believe they are currently infertile and those who believe they have the potential to be infertile later. Fifth, in these cross‐sectional data, we were unable to assess subsequent impacts of perceived infertility. Further research is needed to better understand this phenomenon and how it affects the life course of Ghanaian women.

In Ghana (as in many other contexts), childbearing is viewed as an essential role of women. Many people fear infertility and the social, psychological, economic, and health repercussions that may stem from it. However, there remains a dearth of research on infertility, perceptions around fertility, and the potential impacts of these experiences, particularly in LMICs. Acknowledging the role of infertility (and the urgent need to address infertility in LMICs) as well as the role of perceived infertility is an important component of efforts to support women's ability to decide whether and when to have children. Coupling such efforts with improved contraceptive counseling that addresses any misinformation related to modern methods’ link to infertility, and with education on the likelihood of pregnancy in various scenarios can help ensure that women's reproductive needs and concerns are addressed, while limiting the likelihood of unintended pregnancy.

## DECLARATION OF INTERESTS

We declare no competing interests.

## Data Availability

De‐identified versions of the Community‐Based Survey collected by the authors and used in this analysis are available from the Guttmacher Institute upon reasonable request to researchers who wish to use the data for scholarly analysis. To discuss obtaining copies of these datasets, please contact popcenter@guttmacher.org with the detailed protocol for your proposed study, and information about the funding and resources you have to carry out the study.
